# Concordance studies between hospital discharge data and medical records for the recording of lower extremity amputation and diabetes in the Republic of Ireland

**DOI:** 10.1186/1756-0500-6-148

**Published:** 2013-04-15

**Authors:** Claire M Buckley, Patricia M Kearney, Fawzi Ali, Cliodhna Ni Bhuachalla, Caoimhe Casey, Graham Roberts, Ivan J Perry, Colin P Bradley

**Affiliations:** 1 Department of General Practice, University College Cork, Room 2.57, Western Gateway Building, Cork, Ireland; 2 Department of Epidemiology & Public Health, University College Cork, Cork, Ireland; 3 Department of Medicine and Metabolism, Waterford Institute of Technology, Waterford, Ireland; 4 Department of Microbiology, St James Hospital, Dublin, Ireland

**Keywords:** Concordance study, Lower extremity amputation, Diabetes, Hospital discharge data, Medical records, Agreement statistics

## Abstract

**Background:**

Hospital discharge data have been used to study trends in Lower Extremity Amputation (LEA) rates in people with and without diabetes. The aim of this study was to assess the reliability of routine hospital discharge data in the Republic of Ireland (RoI) for this purpose by determining the level of agreement between hospital discharge data and medical records for both the occurrence of LEA and diagnosis of diabetes.

**Methods:**

Two concordance studies between hospital discharge data (HIPE) and medical records were performed. To determine the level of agreement for LEA occurrence, HIPE records were compared to theatre logbooks in 9 hospitals utilising HIPE over a two-year period in a defined study area. To determine the level of agreement for diabetes diagnosis, HIPE records were compared to laboratory records in each of the 4 largest hospitals utilising HIPE over a one week period in the same study area. The proportions of positive and negative agreement and Cohen’s kappa statistic of agreement were calculated.

**Results:**

During a two-year study period in 9 hospitals, 216 LEAs were recorded in both data sources. Sixteen LEAs were recorded in medical records alone and 25 LEAs were recorded in hospital discharge records alone. The proportion of positive agreement was 0.91 (95% CI 0.88-0.94), the proportion of negative agreement was 0.99 (95% CI 0.98-0.99) and the kappa statistic was 0.91 (95% CI 0.88-0.94).

During a one-week study period in 4 hospitals, 49 patients with diabetes and 716 patients without diabetes were recorded in both data sources. Eighteen patients had diabetes in medical records alone and 2 patients had diabetes in hospital discharge records alone. The proportion of positive agreement was 0.83 (95% CI 0.76-0.9), the proportion of negative agreement was 0.99 (95% CI 0.98-0.99) and the kappa statistic was 0.82 (95% CI 0.75-0.89).

**Conclusions:**

This study detected high levels of agreement between hospital discharge data and medical records for LEA and diabetes in a defined study area. Based on these findings, we suggest that HIPE is sufficiently reliable to monitor trends in LEAs in people with and without diabetes in the RoI.

## Background

Routine hospital discharge data are recognised as an important potential resource for research [1] and it is essential that the data recorded are known to be valid and reliable. Lower Extremity Amputation (LEA) is an established marker of quality of care and data has been published worldwide on the trends of LEAs in people with and without diabetes [2-5]. In Italy, hospital discharge data has been shown to be suitable for the surveillance of LEAs in patients with diabetes [6].

In the Republic of Ireland (RoI), the Hospital Inpatient Enquiry system (HIPE) system is a routine dataset which records administrative, demographical and clinical data on discharges and deaths. Data in HIPE is retrieved from patients’ administration and medical notes [7]. Although not designed as a research tool, HIPE data have been analysed to monitor trends in hospital discharges for various procedures and diagnoses [8-10]. There has been some debate in the literature if routinely collected data in HIPE is sufficiently accurate for research purposes [11,12]. Studies to date on the discharge coding accuracy of HIPE in the RoI are sparse and have reported both under- and over-recording of procedures and diagnoses [13-15].

We have recently reported trends in the incidence of LEAs in people with and without diabetes in the RoI using hospital discharge data [16]. A limitation of our work is that the reliability of these data is not well defined. Thus, the aim of this study was to assess the reliability of routine hospital discharge data in the RoI for research and monitoring of trends in LEA by determining the level of agreement between hospital discharge data and medical records for both the occurrence of LEA and diagnosis of diabetes.

## Methods

This paper describes two concordance studies. Firstly, to determine the level of agreement for LEA occurrence, HIPE records were compared to theatre logbooks. Secondly, to determine the level of agreement for diabetes diagnosis, HIPE records were compared to laboratory records.

### Measurement

HIPE is a computer based health information system that gathers data on discharges from 62 hospitals (60 public and 2 private) in the RoI [11]. The database is managed by the Economic and Social Research Institute (ESRI) [7] and the Health Service Executive (HSE) [17]. Diagnoses and procedures performed as an in-patient or day-patient are recorded and a clinical coder translates the medical terminology in all of the patient’s notes into alpha-numeric codes. Data quality checks are performed to ensure accuracy of data coding [7]. Access to HIPE data is available through Health Atlas Ireland; an open-source software (OSS) mapping, database and statistical system [18].

The study was undertaken in 3 counties (Cork, Kerry and Waterford) in the South of Ireland. For the first concordance study on the procedure of LEA, HIPE data was collected from 9 individual hospitals utilising HIPE for 2 years, 2008–2009. A LEA was defined as complete loss of any part of the lower limb and patients whom underwent LEA during 2008–2009 were identified using the relevant ICD 10 Codes (Blocks 1484, 1505 and 1533) [16,19]. Theatre log books for the same time-period were manually searched for LEAs. Recording styles differed between hospitals; some recorded procedures per consultant and others recorded procedures per theatre. Log-books for all consultants (general, vascular, orthopaedic, plastics) and all theatres (general, vascular, orthopaedic, plastics) were reviewed. All out of service/emergency log-books were also reviewed to ensure all cases were identified. To reduce measurement bias, theatre log books were independently reviewed by two individuals and any discrepancies were reviewed at source. Actual counts of the total number of surgical procedures conducted in the 9 hospitals during the 2-year study period were extracted from HIPE, via Health Atlas, using the relevant ICD-10 codes.

For the second concordance study on the diagnosis of diabetes, all laboratory records of approximately 200 consecutive patients discharged during the first week of December in 2010 from each of the 4 largest hospitals utilising HIPE in the study area were checked. Convenience sampling was used when choosing the hospitals for data collection. A history of a positive Oral Glucose Tolerance Test (OGTT), a Glycosylated haemoglobin (HbA1c) test result of ≥ 6.5% or a random glucose of ≥11.1 mmol/L indicate that a diagnosis of diabetes is likely [20,21] and patients with any one of these 3 findings were assumed to have a diagnosis of diabetes. Diabetes status as per laboratory records was compared to HIPE records. In one hospital, the medical case notes of any patient that had a documented positive OGTT, an HbA1c test result ≥6.5% or a random glucose ≥11.1 mmol/L but did not have a diagnosis of diabetes on the HIPE record were individually checked by an experienced clinician to determine the true diabetes status of the patient.

### Statistical analysis

2×2 tables were constructed for LEA procedures and diabetes diagnoses. Initially, taking medical records as the gold standard, sensitivity, specificity, and positive and negative predictive values (PPV and NPV) were calculated. Sensitivity was calculated as *a/(a + c) × 100%*, specificity as *d/(b + d) × 100%,* PPV as *a/(a + b) × 100%* and NPV as *d/(c + d) × 100%*[22,23].

Given that neither medical records nor HIPE represent a true ‘gold standard’, the proportions of positive and negative agreement and Cohen’s kappa statistic were calculated to quantify agreement rates between findings for the occurrence of LEA and for the diagnosis of diabetes in medical (theatre log-books or laboratory records) and hospital discharge (HIPE) records. The proportion of positive agreement was computed as *PA = 2a/2a + b + c* and the proportion of negative agreement as *NA = 2d/2d + b + c*[22,24]*.* Cohen’s kappa statistic (Ƙ) was equal to (*p*_*o*_*– p*_*e*_*)/ (1 - p*_*e*_*)*, with *p*_*o*_ and *p*_*e*_ the observed and expected agreement by chance, respectively [25-27]. From the 2×2 table, *p*_*o*_*= (a + d)/n* and *p*_*e*_*= [(a + b) (a + c) + (c + d) (b + d)]/n*^*2*^. Analyses were performed using STATA version 12 (Stat Corporation, College Station, Tx, USA).

### Ethical approval

Ethical approval was granted by the CREC (Clinical Research Ethics Committee) of the Cork Teaching Hospitals. As data extracted by the researchers were coded with a Medical Record Number (MRN) around which strict confidentiality safe-guards are in place it was deemed, by the Ethics Committee, that individual patient consent was not required.

## Results

### LEA procedures

Table 1 outlines the distribution of LEA procedures in 9 hospitals utilising HIPE in 2008–2009 in the study area and whether these LEAs were recorded in one or both data sources. Two hundred and sixteen procedures were documented in both data-sources, 16 procedures were recorded in medical records (theatre log-books) alone and 25 procedures in hospital discharge records (HIPE) alone (Table 2). Using the medical records i.e. theatre log-books as the gold standard, HIPE had a sensitivity of 93% (95% CI 90–96), specificity of 100%, PPV of 89% (95% CI 86–92) and NPV of 100%.

**Table 1:** LEA procedures recorded by data source and hospital

**Hospital**	**Hospital discharge & medical records**	**Medical records (theatre log-books) alone**	**Hospital discharge records (HIPE) alone**
I	88	9	14
II	73	4	7
III	17	0	3
IV	3	0	0
V	3	0	0
VI	2	1	0
VII	0	0	0
VIII	0	0	1
IX	30	2	0
**Total**	**216**	**16**	**25**

**Table 2:** Comparison of recording of LEA in HIPE and logbook

	**LEA recorded in log-book**	**LEA not recorded in log-book**	**Total**
LEA recorded in HIPE	216	25	241
LEA not recorded in HIPE	16	306,431	306,447
Total	232	306,456	306,688

Between the two data sources, HIPE and theatre logbooks, the proportion of positive agreement was 0.91 (95% CI 0.88-0.94), the proportion of negative agreement was 0.99 (95% CI 0.98-0.99) and the kappa statistic was 0.91 (95% CI 0.88-0.94) for the occurrence of a LEA procedure.

### Diabetes diagnosis

Table 3 outlines patients with a diagnosis of diabetes from 785 discharges in the first week of December 2010 in the 4 largest hospitals utilising HIPE in this study. Forty-nine patients with diabetes and 716 patients without diabetes were documented in both data sources; 18 patients had diabetes in medical records (laboratory records) alone and 2 patients had diabetes in hospital discharge records (HIPE) alone (Table 4). Using the medical records i.e. laboratory results as the gold standard, HIPE had a sensitivity of 73% (95% CI 66–76), specificity of 99% (95% CI 99–100), PPV of 96% (95% CI 87–99) and NPV of 97% (95% CI 96–98).

**Table 3:** Diabetes diagnosis recorded by data source and hospital

**Hospital**	**Hospital discharge & medical records**	**Medical records (laboratory records) alone**	**Hospital discharge records (HIPE) alone**
I	11	2	1
II	12	4	1
III	11	2	0
IV	15	10	0
**Total**	**49**	**18**	**2**

**Table 4:** Comparison of recording of diabetes diagnosis status in HIPE and laboratory records

	**Diabetes as per lab records**	**No diabetes as per lab records**	**Total**
Diabetes as per HIPE lab records	49	2	51
No diabetes as per HIPE	18	716	734
Total	67	718	785

Between the two data sources, HIPE and laboratory records, the proportion of positive agreement was 0.83 (95% CI 0.76-0.9), the proportion of negative agreement was 0.99 (95% CI 0.98-0.99) and the kappa statistic was 0.82 (95% CI 0.75-0.89) for the diagnosis of diabetes status.

Table 5 outlines results from one hospital which identified 3 patients with conflicting diabetes diagnosis statuses as per HIPE and laboratory records. The first patient had a diagnosis of diabetes documented in both the laboratory and medical notes but not captured by HIPE. The second patient did not have any evidence of diabetes in the laboratory records but had a “once-off” recording of diabetes in the medical notes. The third patient had diabetes as per American Diabetes Association (ADA) HbA1c cut-off points on the laboratory records but a diagnosis of ‘Impaired Glucose Tolerance’ was documented in the medical notes.

**Table 5:** Clinician appraisal of medical notes for patients with conflicting diabetes diagnosis statuses as per HIPE and laboratory records

**Patient number**	**Laboratory record status**	**HIPE status**	**True status as per clinician appraisal**
1	Diabetes	No diabetes	Diabetes
2	No diabetes	Diabetes	No diabetes
3	Diabetes	No diabetes	Diabetes

## Discussion

Routine datasets are commonly used in surveillance [19]. To ensure appropriate use of available datasets, information must be reliable and valid. This study investigates whether HIPE is sufficiently reliable as a data source to use for the purposes of monitoring trends in people with and without diabetes in the RoI. While the findings of this study are relevant to the RoI, the methodological approach applies to the use of routine data anywhere on LEAs in people with and without diabetes.

Initially, we considered the medical records to be the ‘gold standard’ [28,29]. To assess procedure accuracy, HIPE recordings of hospital discharges for LEAs were compared to records of LEAs from a manual search of theatre records. HIPE records demonstrated a PPV of 89% for recording procedures which is comparable to findings from previous systematic reviews of discharge coding accuracy in the UK; 84.2% (Great Britain), 69.5% (England/Wales) and 98% (Scotland) [30,31]. In Italy in 1996, Vaccaro *et al.* compared discharge data to the buried limbs register and reported a procedure discharge coding accuracy of 100% for LEA [6]. Diagnosis accuracy was assessed comparing HIPE records of diabetes status and laboratory records. HIPE records demonstrated a PPV of 96% for recording diabetes status. Again, this is comparable to results from the UK; 80.3% (Great Britain), 91% (England/Wales) and 82% (Scotland) [30,31].

Both systematic reviews performed in the UK and described above compared routine discharge statistics to original medical records. Initially, this study was designed to also compare routine hospital discharge data to medical records and thus, act as a validation study of HIPE on the assumption that medical records represented the ‘gold standard’. However, from review of the literature and findings on field work, it appeared that medical records are not always complete and not all LEA procedures are captured in theatre logbooks [32]. Thus, this study evolved into concordance studies of two data sources (hospital discharge data and medical records). While we have calculated sensitivity, specificity, positive and negative predictive values of routine hospital discharge data compared to medical records to allow comparison with previous research, we recognise that in the absence of a true ‘gold standard’ another measure of agreement was required.

Different techniques exist to measure agreement in concordance studies [22]. The proportion of positive agreement is recommended when only one relationship is the object of interest e.g. agreement between hospital discharge data and medical records [33]. For procedures, we were interested in the agreement between positive findings of LEA in the log-books and HIPE. The proportion of positive agreement of 0.91 (95% CI 0.88-0.94) indicates high agreement between both data sources for LEA.

The kappa statistic is a relative measure that determines the excess of observed agreement to chance agreement and is dependent of the prevalence of the condition [34]. As LEAs are rare events [19], the kappa statistic is largely driven by patients who did not have a LEA recorded in either data source. However, for the diagnosis of diabetes, the kappa statistic is more suitable as the prevalence of diabetes in the adult population in the RoI is approximately 4.5% and continues to rise [35,36]. The kappa statistic of 0.82 (95% CI 0.75-0.89) reflects high levels of agreement of diabetes status between laboratory records and HIPE [37,38].

### Strengths and limitations

Previous studies in the RoI have reported inaccuracies in HIPE recording. O’Callaghan *et al.* reported discrepancies between HIPE records and a prospective vascular database at one site, with HIPE over –recording 4 of 5 studied procedures [15]. Clarke *et al.* reported HIPE underestimating the complexity of discharges in 45 of 100 consecutive stroke patients at one site [13]. Mehanni *et al.* reported a coding accuracy of primary diagnoses of 59% in 793 randomly selected charts at one site [14]. While these studies are extremely informative, agreement of hospital discharge data and medical records at a single site reflects the accuracy of coding at that site. This study includes data from all 9 hospitals utilising HIPE in the study region for the procedure of LEA and data from the 4 largest of those hospitals for the diagnosis of diabetes. Thus, our findings reflect coding accuracy over a defined geographical region and suggest that HIPE may be more reliable than previously reported [13-15]. An overall judgement on the utility of HIPE for research would require investigation of its accuracy for a wider range of procedures and diagnoses and across a wider geographical area.

Best data capture is prospective and/or comes from multiple sources [32,39,40]. The advantage of multiple data sources is that capture rates improve when more than three databases are used [41]. We originally considered including a third data source for LEA records but all potential data sources were deemed ineligible. Considerations included limb fitting records, anaesthetic databases and personal records of healthcare professionals such as podiatrists, diabetic nurse specialists and vascular surgeons. However, many private healthcare professionals in the study region declined an invite to participate in the study and record-keeping styles in the various hospitals involved were inconsistent. The use of only two data sources is a major limitation of this study.

As previously documented, some LEA procedures may not be recorded in the theatre log books [32]. Thus, this study may over-estimate the reliability of HIPE data by comparison to actual clinical activity. Another potential limitation is that the use of laboratory records to detect the status of diabetes diagnosis could be flawed. In one hospital, the full medical notes of patients with conflicting diabetes diagnosis statuses as per HIPE and laboratory records were reviewed by an experienced clinician to ascertain the patient’s true diabetes status. The true status matched the status as per the laboratory records in all 3 patients with conflicting findings. This exercise detected incorrect recordings by clinicians and HIPE staff suggesting that both administration and medical staff contribute to discrepancies between the two data sources. No evidence of a systematic error was detected.

Coding of HIPE data does not occur in real time and completion can take over a year [15]. To ensure timing was not a factor in this study, data from 2008–2009 was gathered in 2011 for procedures and data from 2010 was gathered in 2012 for diagnoses to ensure sufficient time had lapsed to allow HIPE coding to take place.

Measurements of both the procedure of LEA and the diagnosis of diabetes are fraught with pitfalls. LEA reporting methods demonstrate significant variation with no single standard [3]. The debate continues on the use of Oral Glucose Tolerance Tests versus HbA1c assays for the diagnosis of diabetes [21,42]. This study is not directly trying to address these pitfalls. Rather, it assesses if the recording of the procedure of LEA or diagnosis of diabetes concords between hospital discharge data and the medical records.

## Conclusions

In conclusion, results of this study from a defined geographical region in the RoI suggest that hospital discharge data is reasonably accurate in recording the procedure of LEA and the diagnosis of diabetes. There is no consensus of what is acceptable data accuracy [30]. No method is 100% accurate and a margin of error will always exist. This study documented agreement statistics of 0.91 (95% CI 0.88-0.94) for LEA (proportion of positive agreement) and 0.82 (95% CI 0.75-0.89) for diagnoses (kappa statistic). Based on these findings, we suggest that HIPE is sufficiently reliable for use in monitoring trends in LEAs in people with and without diabetes.

### Availability of supporting data

Access to HIPE data is available through Health Atlas Ireland; an OSS mapping, database and statistical system [18]. Special training and permission is required to access this software.

## Abbreviations

ESRI: Economic and social research institute; HbA1c: Glycosylated haemoglobin; HIPE: Hospital In-patient Enquiry; HSE: Health service executive; LEA: Lower extremity amputation; NA: (Proportion of) negative agreement; NPV: Negative predictive value; OGTT: Oral glucose tolerance test; OSS: Open-source software; PA: (Proportion of) positive agreement; PPV: Positive predictive value; RoI: Republic of Ireland.

## Competing interests

This project is partially funded by the HRB (Health Research Board), Ireland – Grant Reference Number: HPF/2009/79 and partially funded by the ICGP (Irish College of General Practitioners) Research and Education Foundation. No potential conflicts of interest relevant to this article are reported.

## Authors’ contributions

CMB conceived the study, analysed the data and wrote the manuscript. PMK conceived the study, interpreted the data, and critically revised the manuscript. FA acquired the data and critically revised the manuscript. CNB acquired the data and critically revised the manuscript. CC acquired the data, interpreted the data and critically revised the manuscript. GR critically revised the manuscript. IJP conceived the study, interpreted the data, and critically revised the manuscript. CPB conceived the study, interpreted the data, and critically revised the manuscript. All authors read and approved the final manuscript.
